# When the Pacemaker Goes Rogue: Pacemaker‐Induced Tachycardia, Syncope, and Car Crash

**DOI:** 10.1155/cric/5546851

**Published:** 2026-05-26

**Authors:** Hanna Grogg, Nikola Kozhuharov, Andreas Haeberlin, Stephan van Gool, Miriam Luginbühl, Mathieu Stadelmann, Bruno Schnegg

**Affiliations:** ^1^ Department of Cardiology, Inselspital, Bern University Hospital and University of Bern, Bern, Switzerland, unibe.ch; ^2^ Department of Intensive Care Medicine, Inselspital, Bern University Hospital and University of Bern, Bern, Switzerland, unibe.ch; ^3^ Department of Cardiology, Spitalzentrum Centre hospitalier Biel-Bienne, Biel, Switzerland

**Keywords:** hemodynamic instability, pacemaker reprogramming, pacemaker-mediated tachycardia (PMT), retrograde conduction, syncope

## Abstract

A 78‐year‐old male with a history of ischemic heart disease with prior myocardial infarction, severe left ventricular systolic dysfunction, and a device‐related implantable cardioverter defibrillator (DR ICD) was admitted following a road traffic accident due to a sudden loss of control of the vehicle following a syncope. Initial investigations ruled out acute coronary syndrome and did not point to neurological causes. Device interrogation revealed the presence of pacemaker‐mediated tachycardia (PMT), but since the episode trigger had been programmed off, it was not possible to determine when they occurred, how long they lasted, or their rate. However, atrial threshold testing promptly provoked a PMT. The induced PMT led to a tachycardia of 130 bpm, causing severe symptomatic hypotension with a mean blood pressure falling below 50 mmHg. This could be easily reproduced several times. Given the patient′s severe left ventricular systolic dysfunction and the absence of any overt alternative explanation, the PMT likely triggered the syncope preceding the car accident. Management focused on reprogramming the DR ICD. The most critical adaptation was a mode switch from DDD to DDIR to prevent atrial tracking, effectively terminating PMT episodes. A prolongation of the postventricular atrial refractory period (PVARP) was not possible due to the very slow VA conduction. The follow‐up revealed no other episodes of PMT and the patient no longer experiences episodes of presyncope or syncope. This report highlights the potential for PMTs to induce severe hemodynamic instability and syncope, underscoring the importance of meticulous device evaluation.

Key Learning Points

• Antidromic near‐field endless‐loop tachycardia (ELT) requires intact retrograde VA conduction with retrograde P waves sensed outside the programmed PVARP, leading to atrial tracking with subsequent ventricular tracking that induces a re‐entry loop at or below upper tracking rate (UTR).
• Although PMT is often benign and automatically terminated by device algorithms, it may become hemodynamically significant in patients with advanced structural heart disease, where tachycardia and loss of atrial contribution to ventricular filling reduce cardiac output.
• Switching from a tracking mode to a nontracking atrial mode (i.e., DDIR) represents a reliable strategy to prevent recurrent PMT when other programming options (i.e., PVARP prolongation) are limited.

## 1. Introduction

**Figure 1:**
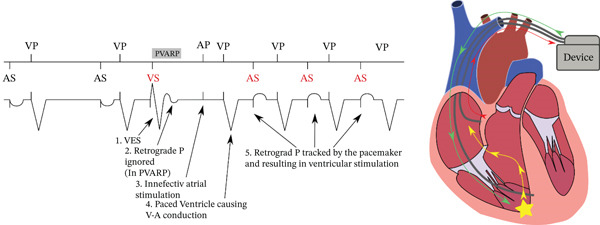
Schematic representation of the mechanism of paradoxical induction of pacemaker‐mediated tachycardia by VPC (ventricular extrasystole). An undetected P wave (sinus or retrograde following VES) renders the subsequent atrial pacing ineffective because the pacing occurs within the atrial myocardial refractory period. The ineffective atrial stimulus causes AV dissociation followed by retrograde VA conduction and initiation of endless‐loop tachycardia.

The implantation of a permanent pacemaker is indicated in patients with atrioventricular (AV) block, whereas an implantable ICD is recommended in patients with ischemic cardiomyopathy and reduced left ventricular ejection fraction for the prevention of sudden cardiac death [1]. A potential consequence of dual‐chamber pacing, in which leads are placed in the right atrium and ventricle, is the occurrence of PMT. This arrhythmia arises from retrograde VA conduction that is tracked by the device, leading to a near‐field ELT. [2] (Figure 1).

Although often self‐limiting or asymptomatic, PMT can be clinically significant in patients with advanced systolic dysfunction. In such cases, loss of atrial contribution to ventricular filling combined with tachycardia may cause a marked reduction in cardiac output and cerebral perfusion [3].

We present the case of a patient with ischemic cardiomyopathy in whom such a mechanism was identified as a likely contributor to syncope.

## 2. Case Presentation

A 78‐year‐old man was referred to our tertiary hospital following a road traffic accident with severe traumatic injuries. Witnesses reported that the patient′s car maintained a constant speed before abruptly veering off course and colliding with a bus shelter without any braking or steering attempts.

Upon arrival, the patient was hemodynamically stable and exhibited no focal neurological deficits, although he had no recollection of the event. In the emergency department, clinicians documented recurrent episodes of tachycardia associated with transient hemodynamic compromise. CT imaging identified multiple intracranial hematomas, a mandibular fracture, and left‐sided rib fractures with pneumothorax. All injuries were managed conservatively with adequate analgesia and scheduled follow‐up imaging.

The patient had a history of ischemic heart disease following an anterior myocardial infarction in 2005, treated with five aortocoronary bypass grafts. He subsequently developed chronic heart failure with severe left ventricular systolic dysfunction (LVEF 30%) and underwent implantation of a dual‐chamber implantable cardioverter‐defibrillator (Abbott/St. Jude Medical, Gallant DR) for secondary prevention after resuscitated ventricular fibrillation in August 2024, with an Optisure LDA210Q‐65 defibrillation lead positioned in the right ventricle and a Tendril STS 2088TC pacing lead in the right atrium.

The patient was receiving guideline‐directed medical therapy for heart failure, including sacubitril/valsartan, spironolactone, metoprolol, and dapagliflozin. In addition, he was treated with atorvastatin and rivaroxaban for paroxysmal atrial fibrillation.

Three days before the accident, the patient experienced two presyncopal episodes, one resulting in a fall and facial hematoma. He described a sudden loss of strength in his legs without prodromal symptoms but did not lose consciousness. He denied chest pain, palpitations, worsening dyspnea, or increased peripheral edema. On the day of the collision, he felt well while driving, and his last memory before the crash was free of any palpitations, dizziness, or chest discomfort.

Troponin levels were slightly elevated without significant dynamic change (20 ng/L at baseline, 22 ng/L after 2 h). The ECG showed sinus rhythm with ventricular‐paced complexes, prolonged AV conduction, and frequent ventricular and supraventricular extrasystoles. Transthoracic echocardiography revealed a severely reduced left ventricular ejection fraction (LVEF < 30*%*) and impaired right ventricular function, both consistent with previous assessments. No significant valvular abnormalities were present. Given the stable findings, neither myocardial contusion nor acute ischemia was suspected. Device interrogation was subsequently performed. A complete system check confirmed normal lead function and revealed complete AV block without a ventricular escape rhythm. But during atrial electrode pacing threshold testing in DDD mode, repeated PMTs were induced, characterized by subthreshold atrial stimulation followed by ventricular pacing and subsequent ventriculoatrial conduction. The onset of these PMTs was marked by immediate and pronounced hemodynamic symptoms, with hypotension observed on the invasive monitoring and the patient reporting dizziness. This prompted close observation of the pacemaker behavior.

**Figure 2:**
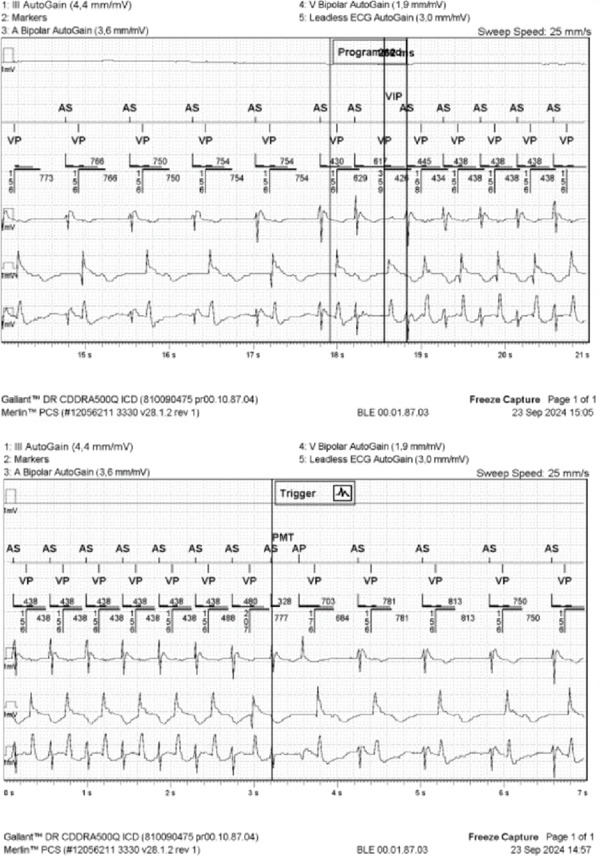
Tachycardia developed due to an active VIP algorithm, resulting in a prolonged AV conduction interval, facilitating intrinsic ventricular activation. Following an initial premature atrial contraction, the long AV delay allowed retrograde conduction, triggering atrial activation with a retrograde P wave. This P wave was then tracked by the DDD mode. The retrograde P wave was misinterpreted as a native sinus P wave, triggering ventricular pacing and initiating a PMT.

These PMT episodes were triggered by premature atrial contraction, followed by a prolonged AV delay due to ventricular intrinsic preference (VIP) programming. In this patient without AV conduction, no intrinsic ventricular activation occurred during the extended delay. At the end of the AV interval, the device delivered a ventricular pacing stimulus, which initiated retrograde conduction to the now excitable atrium. The resulting retrograde P wave was sensed and tracked again by the ICD, perpetuating a near‐field ELT at a rate of approximately 135 bpm (Figure 2).

The documented PMT facilitated by the VIP algorithm underscores the patient′s marked susceptibility to PMTs. However, the high burden of premature ventricular contractions (PVCs) at admission suggests that PVC‐triggered retrograde conduction and subsequent tracking may have played an equally relevant and potentially more frequent role in the occurrence of PMTs.

**Figure 3:**
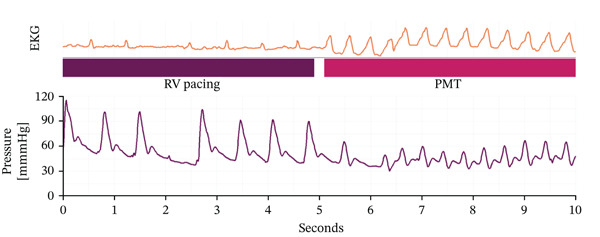
Simultaneous ECG and invasive arterial pressure tracing demonstrating hemodynamic deterioration and visible decline in arterial blood pressure waveform amplitude during PMT.

The hemodynamic impact of these episodes was marked by a notable drop in arterial pressure, with systolic values around 60 mmHg and mean pressures below 50 mmHg (Figure 3). Although invasive monitoring does not allow precise quantification of cardiac output, the combination of hypotension and patient‐reported dizziness indicates a significant reduction in cerebral perfusion. It should be noted that these episodes were not automatically classified as PMT by the device.

In Abbott systems, PMT detection algorithms may generate a substantial number of falsely labeled or clinically nonrelevant episodes, which can lead to rapid memory saturation. For this reason, PMT episode storage is sometimes deactivated in routine clinical practice. In the present case, PMT episode storage had been deactivated prior to admission, which limited retrospective confirmation of spontaneous episodes at the time of the accident.

The mean VA delay was measured at 275 ms, and the PVARP was adjusted accordingly. Although this modification reduced the frequency of PMTs, occasional episodes persisted due to variability in VA conduction and a high PVC burden. Further prolongation of the PVARP beyond 350 ms would likely have suppressed PMTs more effectively; however, this would have reduced the effective UTR to approximately 110 bpm.

In this 78‐year‐old patient, who regularly engages in physically demanding activities such as tennis and daily taekwondo training, such a limitation was considered insufficient to meet anticipated chronotropic requirements and could have resulted in exertional intolerance. Given the presence of reproducible, hemodynamically significant PMTs and the need for a definitive and safe solution, the device was therefore reprogrammed to DDIR mode (60–140 bpm), preventing antegrade tracking of retrograde atrial activity while preserving adequate rate responsiveness through activation of the rate‐response function.

Following device reprogramming, no further episodes of pacemaker‐mediated tachycardia or syncope were observed during the remainder of the hospital stay. In accordance with national driving regulations and given the suspected arrhythmic cause of syncope, the patient was prohibited from driving for 3 months. He resumed driving 4 months after the event and did not report any apprehension related to driving.

Two subsequent outpatient evaluations with his local cardiologist, including device interrogations, demonstrated stable lead parameters with appropriate sensing, impedance, and pacing thresholds. No recurrence of pacemaker‐mediated tachycardia was documented. Transthoracic echocardiography showed persistently severe left ventricular systolic dysfunction, unchanged from prior assessments.

During 18 months of follow‐up, the patient did not experience recurrent syncope or dizziness and did not report any ICD shocks. At follow‐up, he had returned to his previous level of physical activity, including playing tennis twice weekly and engaging in daily taekwondo training.

## 3. Discussion

In the present case, an ELT represents the most probable explanation for the syncopal event leading to the motor vehicle accident. This interpretation is supported by the reproducible induction of hemodynamically significant ELT during device interrogation, the presence of advanced systolic dysfunction rendering the patient particularly vulnerable to tachycardia‐induced low‐output states, and the complete resolution of symptoms following device reprogramming.

Nevertheless, definitive causality cannot be established with absolute certainty. Alternative causes of transient loss of consciousness must be considered. Although no clinical features suggested a primary neurological event, no EEG or cerebral MRI was performed, and a neurological cause therefore cannot be entirely excluded. A vasovagal syncope remains a potential alternative explanation. In addition, a ventricular arrhythmia below the programmed detection threshold cannot be definitively ruled out.

Several types of PMT exist, reflecting distinct mechanisms by which the pacemaker sustains tachycardia (Table 1). Sensor‐mediated PMT results from inappropriate rate increases due to oversensing by activity sensors. Another form, far‐field tachycardia, occurs when ventricular pacing output is erroneously sensed by the atrial channel. The most frequent and classical form, however, is near‐field ELT, which requires intact retrograde VA conduction [2].

**Table 1:** Overview of PMT subtypes and their underlying mechanisms.

PMT subtype	Core mechanism (essentials)
Antidromic near‐field endless‐loop tachycardia	Retrograde VA conduction falls outside PVARP → atrial sensing → ventricular tracking → re‐entry loop at or below upper tracking rate
Antidrome far‐field endless‐loop tachycardia	Ventricular output inappropriately sensed on the opposite channel → false atrial event → ventricular pacing loop
Sensor‐mediated PMT	Overresponsive activity sensor (vibration, hyperventilation, fever, etc.) drives inappropriate rate‐response pacing up to sensor‐defined limit
Atrial tachyarrhythmia tracking PMT	Intrinsic atrial flutter/fibrillation or sinus tachycardia tracked 1:1 (or 2:1) up to programmed upper tracking limit
Pacemaker‐induced atrial re‐entry	Loss of atrial capture or premature pacing initiates intra‐atrial re‐entrant tachycardia that the device then senses and tracks
Myopotential/EMI tracking PMT	Far‐field skeletal activity or electromagnetic noise sensed as atrial events → rapid ventricular pacing
Runaway pacemaker	Hardware malfunction or battery failure causes uncontrolled rapid pacing independent of sensing (device‐driven tachycardia)
Repetitive nonre‐entrant VA synchrony (RNRVAS)	Retrograde P wave falls *within* PVARP (functionally “invisible”) → atrial pacing during atrial refractory phase

In a prospective monocentric study, 30% of patients were found to have VA conduction. Among patients with VA conduction, 20% had one or more episodes of PMT. In this group, this represented 10% of all patients implanted with a pacemaker [3].

In patients with systolic dysfunction, such tachycardias can markedly impair cardiac output by shortening diastolic filling time and abolishing atrial contribution to preload. [4].

Management of ELT focuses on addressing atrial sensing to prevent retrograde conduction. A first step is the deactivation of AV delay–prolonging algorithms (AV‐hysteresis) or a fixed prolonged AV delay used for right ventricular pacing minimization. In the setting of preserved AV conduction, such features are valuable to reduce unnecessary right ventricular pacing and preserve right ventricular function. However, if a complete antegrade AV block develops, this strategy becomes ineffective and may instead promote ELT in the presence of intact retrograde VA conduction.

One strategy to prevent ELT is to deliver an atrial pacing stimulus immediately after a PVC, rendering the atrium refractory when the retrograde impulse arrives and thereby interrupting the re‐entry circuit. The most commonly adopted strategy is to prolong the PVARP, particularly after a PVC, to avoid sensing retrograde P waves. However, this approach is limited by its negative impact on the UTR, as the maximum tracking rate is inversely proportional to the total atrial refractory period (TARP), defined as the sum of the AV delay and PVARP. Since VA conduction times typically range between 250 and 500 ms, and AV delays in older patients can be prolonged 150–200 ms, this can result in a TARP exceeding 500 ms, thereby limiting the UTR to less than 120 bpm or even less.

An alternative is to disable atrial tracking by switching to DDI mode. Although this reliably terminates ELT, it abolishes AV synchrony in patients with preserved atrial activity, resulting in a reduction in cardiac output of approximately 15%–20%. [5].

In selected patients, invasive strategies may be considered, such as ablation of the retrograde conduction.

Importantly, the majority of patients do not require any intervention: When PMTs are asymptomatic and adequately detected and terminated by pacemaker algorithms, no further action is often necessary (Table 2).

**Table 2:** Management strategies for PMT and their associated machanisms and limitations.

Measure	Principle of action	Main limitation/caveat
Programming interventions		
Automatic ELT‐termination algorithms (PMT intervention, anti‐PMT)	Device detects stable VA interval, then delivers a single AV‐shortened beat and/or 500‐ms ARP to break the loop	Not universal; relies on correct sensing of VA interval
Deactivate AV delay–prolonging algorithms (e.g., VIP/MVP and AV‐hysteresis)	Removes long AV delays that let retrograde P waves fall outside PVARP, eliminating the re‐entry trigger	May ↑ RV pacing burden
Extend fixed PVARP	Ensures retrograde P wave is sensed as refractory, inhibiting g atrial tracking	Lowers maximum tracking rate
Automatic PVARP‐after‐PVC (PVC‐response) algorithm	Adds one‐beat PVARP extension or atrial pacing immediately after a PVC to preempt retrograde conduction	Requires vendor‐specific feature to be enabled, not available in all devices
Switch to nontracking mode (DDI/DDIR/VVI)	Removes atrial‐tracking limb, so retrograde atrial activity cannot trigger VP	Loss of AV synchrony, pacemaker syndrome
Nonprogramming interventions		
Magnet application (asynchronous pacing)	Converts device to fixed‐rate pacing, acutely interrupting the re‐entry	Temporary; requires magnet access
AV node or retrograde pathway ablation (in pacemaker‐dependent patients)	Permanently abolishes retrograde VA conduction, eliminating ELT substrate	Invasive; mandates lifelong pacing
Ablation of PVC focus	Removes trigger that initiates ELT in some patients	Only applicable when a single dominant PVC morphology is present, may not abolish other mechanisms

These therapeutic considerations are particularly relevant in patients with advanced cardiac dysfunction. A tachycardia, whatever its origin, reduces ventricular filling time. If this is not compensated—for example by an increase in central venous pressure—it leads to a reduction in preload and, ultimately, cardiac output. In the context of severe systolic heart failure, even a modest increase in heart rate may be sufficient to induce a low‐output state with cerebral hypoperfusion and transient loss of consciousness.

## 4. Conclusion

ELT is often overlooked or regarded as a minor inconvenience, typically appearing as an entry in the arrhythmia log to be cleared during routine device follow‐up. In the vast majority of cases, ELTs are promptly detected and terminated by pacemaker algorithms without clinical consequence. However, in selected patients with severe left ventricular systolic dysfunction, ELT may cause hemodynamically significant deterioration. In such cases, careful device reprogramming—or, in rare situations, AV node or ectopy ablation—may be required to resolve the arrhythmia and restore stability.

**Nomenclature**
ATP: antitachycardia pacing
AS‐VP: atrial sensed–ventricular pacing
AV: atrioventricular
bpm: beat per minute
CT: computed tomography
DDD: dual‐chamber pacing mode
DDI‐R: dual‐chamber inhibited rate‐response pacing mode
DR: device‐related
HFrEF: heart failure with reduced ejection fraction
ICD: implantable cardioverter defibrillator
LVEF: left ventricular ejection fraction
ms: milliseconds
PVARP: postventricular atrial refractory period
PMT: pacemaker‐mediated tachycardia
TARP: total atrial refractory period
TTE: transthoracic echocardiogram
URL: upper rate limit
VA: ventricular–atrial
VIP: ventricular intrinsic preference
VPC: ventricular premature contraction

## Author Contributions

Hanna Grogg: investigation, writing original draft, and project administration. Nikola Kozhuharov: writing review and editing. Andreas Haeberlin: writing review and editing. Stephan van Gool: writing review and editing. Miriam Luginbühl: writing review and editing. Mathieu Stadelmann: writing review and editing. Bruno Schnegg: supervision, formal analysis, and writing review and editing.

## Funding

Open access publishing was facilitated by the Inselspital Universitatsspital Bern, as part of the Wiley ‐ Inselspital Universitatsspital Bern agreement via the Consortium Of Swiss Academic Libraries.

## Disclosure

All patient data, clinical observations, diagnostic findings, and conclusions presented in this manuscript were independently collected, critically assessed, and interpreted by the authors. The authors retain full responsibility for the content of this manuscript.

## Consent

Written informed consent was obtained from the patient for publication of this case report.

## Conflicts of Interest

The following authors declare these conflicts of interest:

Andreas Haeberlin has travel fees/educational grants from Medtronic, Biotronik, Abbott without impact on personal remuneration. He serves as a proctor/consultant for Medtronic and Boston Scientific.

Nikola Kozhuharov has received research grants from the Swiss National Science Foundation (P400PM‐194477 and P5R5PM_210856), Gottfried und Julia Bangerter‐Rhyner‐Stiftung (2020 and 2025), Freiwillige Akademische Gesellschaft, L. & Th. La Roche Stiftung, the European Society of Cardiology, and the European Union/Swiss Secretary for Education, Research and Innovation.

## Data Availability

The data that support the findings of this study are available from the corresponding author upon reasonable request.
